# Low-dose statin treatment increases prostate cancer aggressiveness

**DOI:** 10.18632/oncotarget.22217

**Published:** 2017-10-31

**Authors:** Alfredo Caro-Maldonado, Laura Camacho, Amaia Zabala-Letona, Verónica Torrano, Sonia Fernández-Ruiz, Kepa Zamacola-Bascaran, Leire Arreal, Lorea Valcárcel-Jiménez, Natalia Martín-Martín, Juana M. Flores, Ana R. Cortazar, Patricia Zúñiga-García, Amaia Arruabarrena-Aristorena, Fabienne Guillaumond, Diana Cabrera, Juan M. Falcón-Perez, Ana M. Aransay, Antonio Gomez-Muñoz, Mireia Olivan, Juan Morote, Arkaitz Carracedo

**Affiliations:** ^1^ CIC bioGUNE, Bizkaia Technology Park, Derio, Spain; ^2^ Biochemistry and Molecular Biology Department, University of the Basque Country, Bilbao, Spain; ^3^ CIBERONC, Madrid, Spain; ^4^ Department of Animal Medicine and Surgery, School of Veterinary Medicine, Complutense University of Madrid, Madrid, Spain; ^5^ Centre de Recherche en Cancérologie de Marseille, U1068, Institut National de la Santé et de la Recherche Médicale, Paris, France; ^6^ Institut Paoli-Calmettes, Marseille, France; ^7^ UMR 7258, Centre National de la Recherche Scientifique, Paris, France; ^8^ Université Aix-Marseille, Marseille, France; ^9^ Centro de Investigación Biomédica en Red de Enfermedades Hepáticas y Digestivas, Madrid, Spain; ^10^ IKERBASQUE, Basque foundation for science, Bilbao, Spain; ^11^ Department of Urology and Research Group in Urology, Vall d´Hebron Hospital, Vall d´Hebron Research Institute, and Universitat Autònoma de Barcelona, Barcelona, Spain

**Keywords:** prostate cancer, statins, cholesterol, obesity, mouse models

## Abstract

Prostate cancer is diagnosed late in life, when co-morbidities are frequent. Among them, hypertension, hypercholesterolemia, diabetes or metabolic syndrome exhibit an elevated incidence. In turn, prostate cancer patients frequently undergo chronic pharmacological treatments that could alter disease initiation, progression and therapy response. Here we show that treatment with anti-cholesterolemic drugs, statins, at doses achieved in patients, enhance the pro-tumorigenic activity of obesogenic diets. In addition, the use of a mouse model of prostate cancer and human prostate cancer xenografts revealed that *in vivo* simvastatin administration alone increases prostate cancer aggressiveness. *In vitro* cell line systems supported the notion that this phenomenon occurs, at least in part, through the direct action on cancer cells of low doses of statins, in range of what is observed in human plasma. In sum, our results reveal a prostate cancer experimental system where statins exhibit an undesirable effect, and warrant further research to address the relevance and implications of this observation in human prostate cancer.

## INTRODUCTION

The initiation, progression and therapeutic eradication of cancer is largely associated to the evolving mutational landscape of the tumor [1]. However, tissue-specific factors, the tumor microenvironment and the immune activation status are determinant factors of cancer cell survival and progression [2]. Critically, systemic metabolic alterations, nutrition, obesity and comorbidity-derived long-term therapies in elderly population shape the incidence and aggressiveness of cancer in our society [3–6]. Prostate cancer (PCa) is among the most frequent tumor type in men, and the main risk factors include family history, race and age [7]. Importantly, due to its predominant diagnosis in men above 60 years old, comorbidities are frequent. These include obesity, metabolic syndrome, arterial hypertension and diabetes [8]. In turn, PCa patients at the time of diagnosis are commonly subject to chronic therapies. Anti-hypercholesterolemic treatment is prescribed to millions of individuals around the globe in the form of statins, and their benefits and harms have been studied [9]. Due to their extensive and chronic use, it is of the utmost importance to carefully evaluate the impact of this long-term therapy on the biology of cancer, at doses and administration modes achieved in human subjects. In this study, we evaluated the impact of statin treatment in the pathogenesis and progression of PCa. Through the use of PCa mouse models, cellular systems and observational studies in patients we demonstrate that treatment with these compounds is associated to increased aggressive features in this disease.

## RESULTS

**Figure 1:**
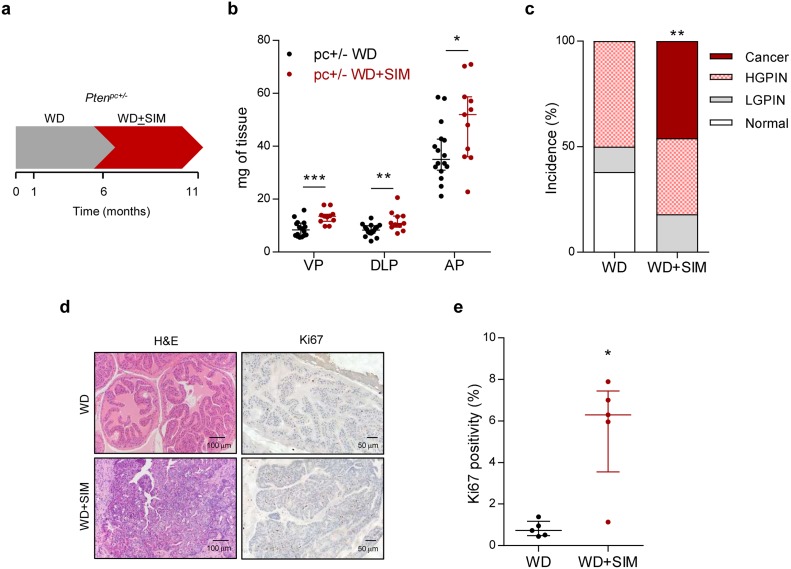
Simvastatin administration cooperates with obesogenic diets to drive prostate cancer **a**. Schematic representation of the experimental design. 4-6 week-old prostate-specific *Pten*-heterozygous (*Pten^pc+/-^*; *pc+/-*) mice were fed with western diet (WD) to induce obesity. At 6 months of age, mice were randomly assigned to WD or WD loaded with simvastatin (WD + SIM) and fed for 5 months, and tissues were harvested and analysed. **b**. Prostate lobes weights of *Pten*-heterozygous (*Pten^pc+/-^*; *pc+/-*) mice fed with WD (*n* = 16) or WD + SIM (*n* = 11). VP, DLP, AP refer to ventral, dorsolateral and anterior prostates, respectively. **c**. Histopathological characterization of the prostate (Normal: no lesions; LGPIN: Low-grade prostatic intraepithelial neoplasia; HGPIN: High-grade prostatic intraepithelial neoplasia; Cancer: prostate adenocarcinoma) (WD, *n* = 16, WD+SIM, *n* = 11). **d**. Representative histological images of the prostate. Left, H&E (Haematoxylin-eosin) and right, Ki67 staining. WD shows non-cancerous tissue, WD+SIM shows adenocarcinoma. **e**. Quantification of Ki67 positive nuclei (%), indicating proliferating cells (WD, *n* = 5; WD SIM, *n* = 5). Statistical analysis: Mann-Whitney statistic test (b, e) and Chi Square test with 3 degree freedom (c). Error bars represent median with interquartile range. *p < 0.05, **p < 0.01, ***p < 0.001.

Anti-hypercholesterolemic treatments are often prescribed in the context of overweight or obesity [16]. Therefore, in order to evaluate the impact of statins (the main family of anticholesterolemic compounds, inhibitors of the mevalonate pathway enzyme Hydroxymethyl glutaryl CoA reductase - HMGCR) in PCa biology, we first evaluated the effect of statin exposure in the context of obesity in *Pten* prostate-specific heterozygous mice (*Pten^pc+/-^*), which exhibit a weak, non-cancerous phenotype [10, 17]. We focused on the predominant statin used in the clinic, simvastatin. It is worth noting that the hydrophilic nature differs among statins, which could lead to distinct biological consequences *in vivo* [18]. We queried the available bibliography (Supplementary Table 1) and determined a low dose of simvastatin with proven biological activity [19]. Simvastatin was loaded in food pellets and provided *ad libitum,* thus enabling the uptake of simvastatin in an administration mode and concentration comparable to human subjects, including the activation of the drug in the liver [20]. We established an experimental design in which we induced obesity by feeding the mice with western diet (containing high fat and carbohydrates) [21], and once the obesity was achieved, simvastatin was loaded to the obesogenic diet (Figure 1a for experimental design). At the end of the experiment, the weight of the mice on western diet was significantly increased compared to mice on standard diet (45.44 ± 1.34 gr *versus* 40.72 ± 2.15 gr, *p* = 0.04), whereas simvastatin addition to western diet did not have an impact on this parameter (46.83 ± 1.05 gr, *p* = 0.43). The combination of obesity and statin treatment increased prostate mass (Figure 1b). At the histological level, obese *Pten^pc+/-^* mice exhibited 50% incidence of prostate intraepithelial neoplasia (PIN) at 11 months of age, without the appearance of invasive carcinoma lesions (Figure 1c, d). Strikingly, simvastatin treatment administered after the onset of obesity (Figure 1a) led to invasive cancerous lesions with an incidence of 45%, a phenotype only achieved in this mouse model when both copies of *Pten* are lost in the prostate epithelium [10, 13] (Figure 1c, 1d). Molecular analysis of these prostates revealed that simvastatin treatment exacerbated cell proliferation, accounted by Ki67 positivity (Figure 1d, 1e). These results provide unprecedented evidence for an undesired consequence of the treatment with statins in obese individuals with genetic predisposition to develop PCa.

**Figure 2:**
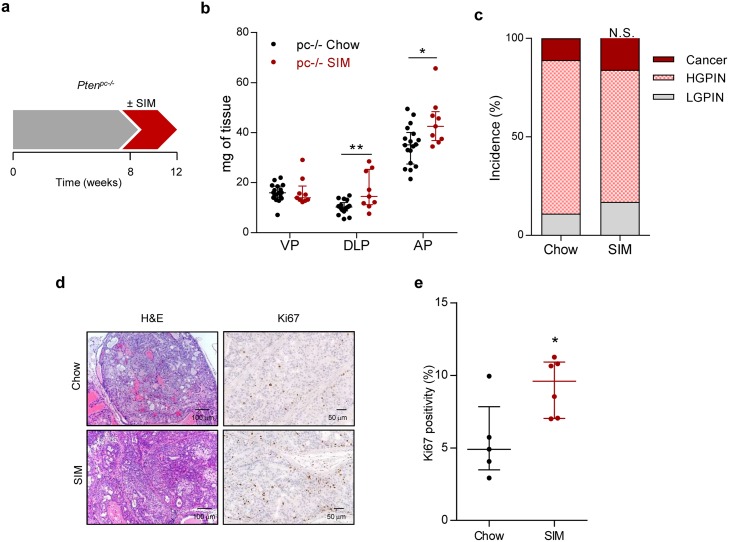
Simvastatin administration increases feature of aggressiveness in prostate cancer initiation **a**. Schematic representation of the experimental design. 8 week-old *Pten*-deficient (*Pten^pc-/-^*; *pc-/-*) mice were fed with simvastatin-loaded (SIM) diet or chow for four weeks, and tissues were harvested and analysed. **b**. Prostate lobe weights of *Pten*-deficient mice fed chow (*pc-/-* Chow, *n* = 18) and SIM diet (*pc-/-* SIM, *n* = 9). VP, DLP, AP refer to ventral, dorsolateral and anterior prostates respectively. **c**. Histopathological characterization of the prostate. (LGPIN: Low-grade prostatic intraepithelial neoplasia; HGPIN: High-grade prostatic intraepithelial neoplasia; Cancer: prostate adenocarcinoma) (Chow, *n* = 9; SIM, *n* = 6). **d**. Representative histological images of the prostate. Left, H&E (Haematoxylin-Eosin) and right, Ki67 staining, showing prostate intraepithelial neoplasia (PIN) in *Pten*-deficient mice fed with SIM or chow. **e**. Quantification of Ki67 positive nuclei (%), indicating proliferating cells, in *pc-/-* Chow (*n* = 5) and *pc-/-* SIM (*n* = 6). Statistical analysis: Mann-Whitney statistic test (b, e) and Chi Square test with 2 degree freedom (c). Error bars represent median with interquartile range. N.S.: Non-significant. *p < 0.05, **p < 0.01.

We next evaluated the impact of statin treatment on PCa initiation using a *Pten* prostate-specific knockout (*Pten^pc-/-^*), which allows the study of proliferative burst under oncogenic signalling [13, 17]. Four-week statin treatment in *Pten^pc-/-^* at the time of *Pten* excision and disease onset (8 weeks of age) resulted in an increased prostate mass and proliferation, without overt histological changes (Figure 2).

PCa initiation, progression and resistance to therapy depend on distinct molecular mechanisms. Advanced PCa is currently treated with androgen-deprivation therapy [22]. Previous reports have documented that *Pten^pc-/-^* mice subject to orchiectomy exhibit overall pathological response [23]. Therefore, we performed surgical castration in order to address effects of statins on cancer cell biology beyond proliferation (Supplementary Figure 1a). In line with the undesirable effect of statin treatment observed in the other experimental systems, we observed a trend towards increased prostate mass and castration resistance in simvastatin-treated mice (Supplementary Figure 1b-d), in the absence of a significant alteration in cell proliferation (Supplementary Figure 1d, e).

In sum, the use of a faithful mouse model of PCa uncovers an unexpected effect of simvastatin that is associated to the increase of PCa cell proliferation, cancer initiation and resistance to therapy.

**Figure 3:**
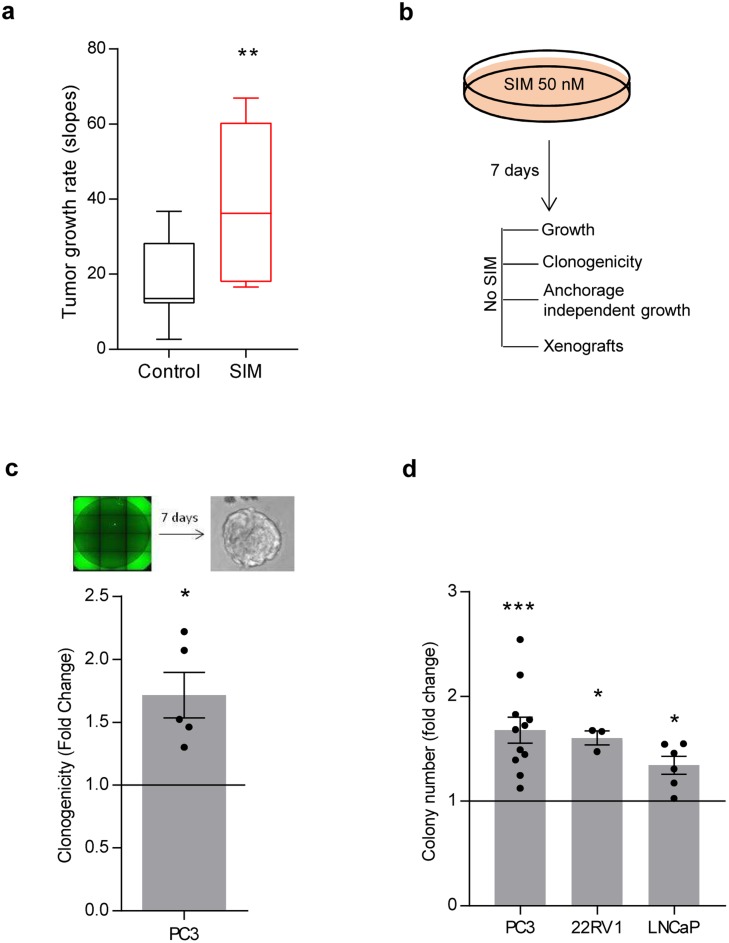
Low-dose simvastatin enhances features of prostate cancer aggressiveness *in vitro* and *in vivo* **a**. Tumor growth rate from PC3 cell xenografts upon feeding nude mice with chow or simvastatin-loaded (SIM) diet. 5 mice for condition were used, 4 tumors per mouse. 100.000 PC3 cells were injected. Mice were fed with simvastatin loaded chow starting 72h before injections. Box plot representation. **b**. Schematic representation of simvastatin treatment *in vitro* in PC3 cells. Cells were pre-treated for 7 days with 50 nM simvastatin, and biological effects were evaluated. **c**. Effect of simvastatin pre-treatment on clonal self-renewal capacity (*n* = 5) in PC3 cells. **d**, Effect of 50 nM simvastatin pre-treatment on anchorage-independent growth in PC3, 22RV1 and LNCaP cell lines. Statistical analysis: Mann Whitney test (a), one sample t test (c, d. Error bars represent standard error of the mean. *p < 0.05, **p < 0.01, ***p < 0.001.

Our data provides evidence of the undesirable effect of simvastatin in murine PCa. To extrapolate these results to human PCa, we took advantage of a human PCa cell line, PC3, and evaluated the impact of simvastatin feeding on tumor growth in subcutaneous xenografts. In line with our previous results, simvastatin fed mice exhibited significantly heightened tumor growth rate (Figure 3a).

Multiple reports have documented the antitumoral effect of statins *in vitro*. The majority of these studies rely on doses of these compounds in the micromolar range [24–45] (Supplementary Table 2). However, the concentration of statins found in plasma of patients subject to anti-cholesterolemic treatments is in the nM range [46]. Therefore, we sought to establish an *in vitro* experimental model that would recapitulate the concentration of statins achieved in patients. To this end, we first corroborated the reported effects of high simvastatin doses. Micromolar doses of simvastatin elicited an anti-proliferative and cytotoxic response in PCa cells (Supplementary Table 2; Supplementary Figure 2a, b). Next, we designed an experimental approach aimed at ascertaining the cell autonomous biological consequences of low simvastatin doses in PCa cells. We pre-treated PC3 cells with 50 nM simvastatin for a minimum of 7 days, which did not result in any sign of toxicity in two-dimensional growth assays (Figure 3b, Supplementary Figure 2c). This treatment schedule did not affect cell migration (Supplementary Figure 2d), but surprisingly resulted in elevated self-renewal capacity and anchorage-independent growth (Figure 3c, 3d). Of note, the effect of simvastatin *in vitro* was recapitulated in other cell lines (LNCaP and 22RV1, Figure 3d) and with an alternative HMGCR inhibitor (fluvastatin, Supplementary Figure 2e). Moreover, moderate genetic inhibition of HMGCR with two independent doxycycline-inducible shRNAs (Supplementary Figure 2f, g) elicited an effect comparable to simvastatin in anchorage-independent growth. Conversely, subtle HMGCR over-expression elicited the predicted opposing effect (Supplementary Figure 2f, h).

Since anchorage-independent growth and self-renewal capacity are required steps in tumor re-initiation and metastatic seeding [47], we evaluated whether PC3 cells treated for 7 days with 50 nM simvastatin would harbour elevated metastatic capacity. To this end, we injected PC3 cells pre-treated with vehicle or simvastatin in the tail vein of immunocompromised mice, and analysed the appearance of lung metastatic lesions (Supplementary Figure 2i right). Simvastatin treatment did not reduce metastatic burden, but rather resulted in increased rate of lung metastasis, which did not reach statistical significance (*p* = 0.1) probably owing to the low number of mice (Supplementary Figure 2i). In sum, our results support the existence of a biological context where statin treatment could promote features of PCa aggressiveness.

The effect of statins could be associated to the alteration in major oncogenic signalling pathways sustaining PCa function. Therefore, we evaluated the levels and/or activity of androgen receptor (AR), Phosphoinositide 3-kinase (PI3K, using as a readout serine 473 phosphorylation of AKT) and mitogen activated protein kinase (MAPK, using as a readout tyrosine 202/204 phosphorylation of ERK - extracellular signal regulated kinase) in AR-expressing LNCaP (AR-dependent) and 22RV1 (AR-independent) cell lines. None of these parameters (AR protein levels or activity by means of the mRNA abundance of its target *KLK3*; AKT or ERK phosphorylation) was consistently altered neither *in vitro* nor *in vivo*, thus precluding their involvement as a major component of the mechanism of action of statins (Supplementary Figure 3; unprocessed scans in Supplementary Figure 5). Of note, we also monitored the expression of cholesterol transporters and metabolic enzymes that could be altered as a consequence of statin treatment [48, 49]. We did not observe consistent changes neither in low density lipoprotein receptor (LDLR) nor in other enzymes and transporters (Apolipoprotein (APO) genes, ACAT1 (acetyl CoA cholesterol acyl transferase) and 2 and lipoprotein lipase (LPL)), precluding a major involvement of such alterations in the biological effect of these compounds (Supplementary Figure 4).

Observational studies have evaluated the association of statins to PCa risk and aggressiveness (Supplementary Table 3) [50, 51]. Our data suggests that there could be a subset of PCa patients where statins could exert undesirable effects. We analyzed data from a prospective study conducted in Vall d´Hebron Research Institute [15]. To carry out this analysis, 2408 men were selected, after excluding those men who were undergoing 5-α-reductase inhibitors treatment and those men who had been using statins for less than three years. In this cohort, the impact of statins was previously evaluated as part of a multivariate analysis with other factors such as age, PSA or serum cholesterol levels; or in combination with plasma cholesterol or aspirin treatment [15, 40]. 37.2% of patients passed the statin treatment criteria beyond 3 years. In the multivariate study, statins were not associated to PCa risk. PCa was detected in 848 men (35.2%), and 297 of them (35%) were classified as high grade (HGPCa, Gleason score >7; as compared to Low Grade PCa (LGPCa; Gleason score ≤7)). In line with previous reports, treatment with this family of compounds reduced the risk of suffering from PCa (overall risk (OR) = 0.717; *p* = 0.006). However, 41.8% of patients treated with statins were diagnosed with HGPCa, whereas aggressive disease was less prevalent among patients not taking the cholesterol-lowering drug (32.5% of statin non-treated patients presented HGPCa, *p* = 0.012, OR 1.495 (1095-2.039)) (Supplementary Table 4). To which extent the effect of statins is a predominant selective effect reducing the incidence of LGPCa, or whether it has an activity promoting the appearance of HGPCa remains to be studied, since both scenarios could lead to the results obtained in our analysis. In addition, our observational study does not account for statin dose, which, according to our experimental data, could be an important factor. Of note, these results are in line with the increased risk of high grade cancer reported in patients subject to statins that show normalized serum cholesterol levels [15], or patients with combined treatment of statins and aspirin [40]. Importantly, these results were corroborated in a multivariate analysis with other chronic pharmacological treatments (Supplementary Table 5). It should be noted that the controversy in observational studies with statins remains high [52], and additional analysis in well-annotated cohorts is granted.

## DISCUSSION

Systemic metabolic alterations impact on the function and cross-talk of cells in our body. Indeed, the incidence and aggressiveness of cancer is in part associated to non-cell autonomous factors, such as nutrition, obesity or chronic therapeutic regimes [3, 53]. Statins are administered to millions of people worldwide. In turn, their consequences on tumor biology have become a research field of great interest. We have studied the consequences of statin treatment in PCa biology, and demonstrated through the use of a wide array of pre-clinical and experimental approaches that these compounds promote features of disease aggressiveness. It should be noted that our experimental systems might reflect the existence of a sub-population of PCa patients for whom statins have undesirable effects, in line with other studies [54, 55]. Interestingly, a large study of 47294 individuals focused on the study of coronary heart disease observed that patients treated with low-dose simvastatin (5-10 mg/day) and achieving low total serum cholesterol, presented increased risk of developing cancer (OR = 3.16 for serum cholesterol < 160; OR = 1.85 for serum cholesterol = 160-179) [56]. The analysis of the available observational studies supports the existence of a context where statins could increase the aggressiveness of the disease in PCa patients. Overall, current epidemiological studies [15, 40, 50, 51, 54–68] would benefit of re-analysis taking into account this new information.

Experimental cancer systems often serve for the validation and causal demonstration of observations originated in patient studies. However, these approaches can also be employed to predict the consequences of societal or lifestyle changes. Our experimental systems provide very provocative results that still lack full clinical validation to demonstrate the potential existence of a subset of PCa patients in which statins exert an undesired activity. Our results warrant further analysis of the cell autonomous and systemic impact of statin treatment in PCa and other cancers in order to understand the biological context associated to a protective or detrimental activity of these compounds.

## MATERIALS AND METHODS

### Cellular and molecular assays

Human prostate carcinoma cell lines (PC3, LNCaP and 22RV1) were purchased from Leibniz-Institut - Deutsche Sammlung von Mikroorganismen und Zellkulturen GmbH (DSMZ, Germany), who provided authentication certificate. In addition, we validated their identity by microsatellite analysis. Cell lines were routinely monitored for mycoplasma contamination. Simvastatin and mevalonate (Sigma-Aldrich) for *in vitro* purposes were activated by heating (50 °C) with NaOH (0.1N) for two hours. Fluvastatin (Sigma-Aldrich) was used following manufacturers’ indications.

For clonogenicity assay, PC3 cells expressing GFP were plated in poly-HEMA pretreated 384 plates at 1 cell per well in DMEM/F12 (Gibco) plus EGF (100 mg/ml), bFGF (10 mg/ml), B27 (Thermo Fisher), 8% BSA (Sigma-Aldrich). Wells with 0, 1, or >1 cells were annotated. 7 days after plating, sphere formation from wells with single cells was quantified. Crystal violet-based cell number quantification, soft-agar anchorage independent growth and western blotting were performed as previously described [10]. Antibodies used for western blotting: androgen receptor (clone D6F11, Cell Signaling #5153), phosphorylated ERK (T202/204; extracellular signal regulated kinase, clone 20G11, Cell Signaling #4376), phosphorylated AKT (S473; clone 736E11, Cell Signaling #3787), ERK (clone 3A7, Cell Signalling #9107), AKT (Cell Signalling #9272), β-Actin (clone AC-74; Sigma #A5316), LDLR (EP1553Y; Abcam #ab52818) and HSP90 (Heat Shock Protein 90, Cell Signaling #4874).

RNA was extracted using NucleoSpin^®^ RNA isolation kit from Macherey-Nagel (ref: 740955.240C). For RNA harvesting from mouse tissue, we introduced a prior step consisting on the incubation of the tissue in RNAlater ICE (Thermo Fisher) overnight at -20°C and phenolic extraction with TRIreagent (TR118, MRC). In all cases, 1μg of total RNA was used for cDNA synthesis using qScript cDNA Supermix from Quanta (ref. 95048). Quantitative Real Time PCR (qRTPCR) was performed as previously described [10]. Universal Probe Library (Roche) primers and probes employed in human samples: *HMGCR*, primers: Fw: gttcggtggcctctagtgag, Rv: gcattcgaaaaagtcttgacaac; Probe: 65. *KLK3*, primers: Fw: gtgcttgtggcctctcgt Rv: agcaagatcacgcttttgttc; Probe: 44. *LDLR*, primers: Fw: gatagtgacaatgtctcaccaagc, Rv: cctcacgctactgggcttc; Probe: 6. *APOD*, primers: Fw: gagaggccagtcaccaagac, Rv: gagaagggacctggagcttt; Probe: 8. *APOA2*, primers: Fw: gagaaggtcaagagcccaga, Rv: ccttcttgatcaggggtgtc; Probe: 68. *APOC1*, primers: Fw: gccttggataagctgaagga, Rv: gaaatgtctctgaaaaccactcc; Probe: 47. *LPL*, primers: Fw: caggcctttgagatttctctgt, Rv: gaaggagtaggtcttatttgtggaa; Probe: 13. Universal Probe Library (Roche) primers and probes employed for mice: *Ldlr*, primers: Fw: gatggctatacctacccctcaa, Rv: tgctcatgccacatcgtc; Probe: 64. *ApoD,* primers Fw: aatttccatcttgggaaatgc, Rv: ggatcttctcaatttcgtaccatc; Probe: 63. *ApoC1,* primers *Fw:* tgggaacactttggaagaca, *Rv:* actttgccaaatgcctctga; Probe: 46. *ApoA2*, primers Fw: tgctggtcaccatctgtagc, Rv: catatccggtccgtctgc; Probe: 12. *ApoE*, primers Fw: ttggtcacattgctgacagg, Rv: agcgcaggtaatcccagaa; Probe: 32. *Lpl*, Fw: tttgtgaaatgccatgacaag, Rv: cagatgctttcttctcttgtttgt; Probe: 47. *Acat1*, Fw: ggctgaactcagtaaccacaca, Rv: ttggcttctagccgattcc; Probe: 71. *Acat2*, Fw: attccagccataaagcaagc, Rv: tttagctattgccgcagaca; Probe: 88. *β-ACTIN* and *GAPDH* housekeeping assays from Applied Biosystems (*β-ACTIN*, Hs99999903_m1; *GAPDH*, Hs02758991_g1; Mm99999915_g1); showed similar results (all qRTPCR data presented was normalized using *GAPDH*).

Lentiviral shRNA sequences targeting HMGCR (TRCN0000262852 and TRCN0000262856, Sigma Mission Library) were cloned into TET-pLKO puro vector (gift from Dr. Dmitri Wiederschain [11], Addgene plasmid #21915). HMGCR over-expressing lentiviral plasmid (pLX304) was obtained from https://plasmid.med.harvard.edu (HsCD00412328).

### Animals

All mouse experiments were carried out following the ethical guidelines established by the Biosafety and Welfare Committee at CIC bioGUNE. The procedures employed were carried out following the recommendations from AAALAC. Xenograft experiments were performed as previously described [12], injecting 10^5^ cells per condition in two flanks per mouse (male Hsd:Athymic Nude-Foxn1 nu/nu). For metastasis experiment, 6×10^5^ cells in 200 μl were injected by tail vein [12]. When possible, mice were injected randomly and xenografts measured blindly to reduce bias due to caging. Western diet (SSNIFF, D12079 mod.) with high carbohydrates and fat (50% carbohydrates, 21% fat) was compared with the 4% fat of the control diet. Simvastatin was provided in the diets (both standard diet and western diet) at 12 mg/kg chow. The supplied concentration of simvastatin in mice was equivalent to 12 mg/day in humans, which corresponded to low doses for anticholesterolemic treatment (the standard dose being 40 mg/day).

The *Pten^loxP^* conditional knockout alleles have been described elsewhere [13]. Prostate epithelium specific deletion was effected by the Pb-Cre4. Mice were fasted for 6 h prior to tissue harvest in order to prevent metabolic alterations due to immediate food intake.

### Histopathological analysis

Samples of prostate gland or lungs were fixed overnight in 10% neutral buffered formalin, embedded in paraffin and sectioned 5 μm thick and dried. Slides were dewaxed and re-hydrated through a series of graded ethanol until water and were stained with hematoxilin-eosin (H-E). Histopathological lesions of the prostate were classified according to current histological criteria [14].

Detection of PC3 in lungs metastatic foci of immunocompromised mice in the metastasis assay was assessed by immunohistochemical staining of Vimentin (NCL-L-VIM-572, Leica biosystems) using the streptavidin-biotin-peroxidase complex.

Proliferation was evaluated in paraffin embedded prostate samples by using Ki67 antibody (MA5-14520, Thermo Scientific). Five fields, at least 400 cells each field from the AP (anterior prostate) lobe were counted.

Immunohistochemical stainings of AR, pERK, pAKT and LDLR (references as in western blot analysis) were performed on deparaffinized prostate sections using the streptavidin-biotin-complex peroxidase method. Antigen retrieval was carried out by heating sections in 10 mM sodium citrate, pH 6.0. Immunodetection was performed with the Polink-2 HRP Plus Rabbit Detection System (D39-110, GBI Labs, Bothell, WA, USA) and slides were developed with the peroxidase substrate kit (SK-4105, Vector Laboratories, Burlingame, CA, USA). Staining score 0 to 3 was given by two independent investigators based on the % of stained cells and the intensity of the staining.

### Patients

We analysed data from a prospective study conducted in Vall d’Hebron Research Institute [15]. To carry out this analysis, 2408 men were selected, after excluding those men who were undergoing 5-α-reductase inhibitors treatment and those men who had been using statins for less than three years. Prostate cancer (PCa) was detected in 848 men (35.2%), and 240 (28.3%) were high grade prostate cancer (HGPCa) (Gleason score > 7). The overall demographics and clinical characteristics of the men enrolled, as well as the methodological aspects have been previously reported [15].

### Statistics

For patient analysis, quantitative variables were expressed as medians + semi-interquartile range. Qualitative variables were expressed as rates. Univariate analysis included the Chi-square test to analyse the association between qualitative variables and the Cochran test to evaluate their strength. Multivariate analysis using the binary logistic regression was carried out to examine the independent predictors of PCa risk and tumor aggressiveness. Odds ratios (OR) and 95% coefficient interval (CI) were calculated.

For *in vivo* studies, in the absence of normal distribution, a non-parametric Mann Whitney U test was applied for two-group comparisons. For frequency analysis, Chi-square test was used when there were more than 2 variables and Fisher F test was used for 2 variables. For *in vitro* experiments, normal distribution was assumed and one sample t-test was applied for one component comparisons with control. Error bars represent mean ± SEM (standard error of the mean) *in vitro*, and median with interquartile range *in vivo*, unless otherwise specified. We considered *p* < 0.05 to be statistically significant.

## SUPPLEMENTARY MATERIALS FIGURES AND TABLES












