# Supplementary material for: Low-dose statin treatment increases prostate cancer aggressiveness
Source: Oncotarget. 2017 Oct 31;9(2):1494–504. doi: 10.18632/oncotarget.22217 (PMC5788577; doi:10.18632/oncotarget.22217)
Supplement: Supplementary file 1 [file oncotarget-09-1494-s001.pdf]

Low-dose statin treatment increases prostate cancer aggressiveness

SUPPLEMENTARY MATERIALS

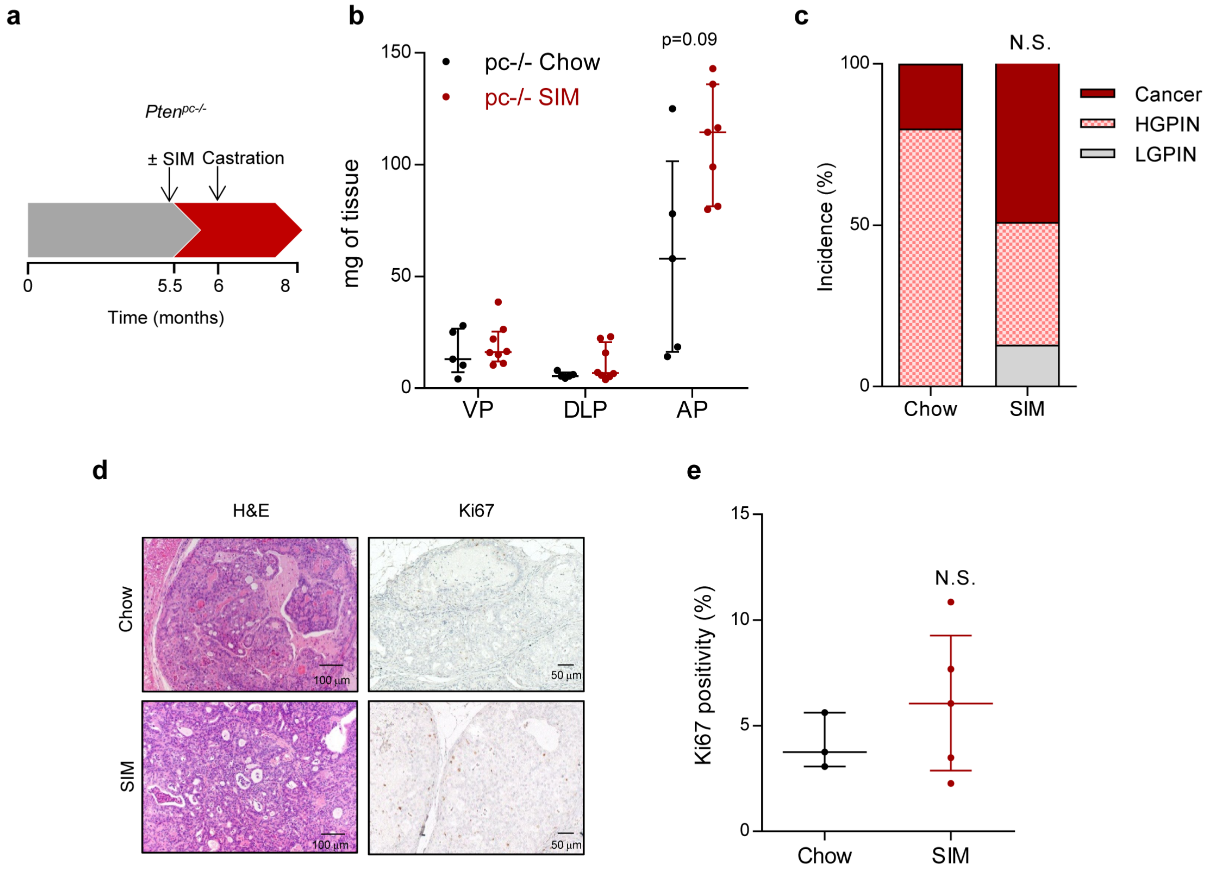

Supplementary Figure 1: (**a**) Schematic representation of the experimental design. 5.5 month-old prostate-specific *Pten*-deficient (*Pten*$^{pc-/-}$; *pc-/-*) mice were fed with simvastatin loaded (SIM) or chow diet for 2 weeks, were then castrated and maintained in the dietary regime for 8 weeks until histological analysis. (**b**) Prostate lobe weight in castrated *Pten*$^{pc-/-}$ mice fed with SIM (n=8) or chow diet (n=5). VP, DLP, AP refer to ventral, dorsolateral and anterior prostates respectively. (**c**) Histopathological characterization of the prostate in SIM (n=8) or chow (n=5) (LGPIN: Low-grade prostatic intraepithelial neoplasia; HGPIN: High-grade prostatic intraepithelial neoplasia; Cancer: prostate adenocarcinoma). (**d**) Representative histological images of the prostate in castrated *Pten*$^{pc-/-}$ mice fed with SIM or chow. From left to right, H&E (Haematoxylin-eosin) and Ki67 staining. Chow shows a castration responder example and SIM shows non-responder (castration-resistant, adenocarcinoma) example. (**e**) Representation of the percentage of Ki67 positive nuclei, indicating proliferating cells (Chow, n=3, SIM, n=5). Statistical analysis: Mann-Whitney test (b, e), Chi Square test with 2 degree freedom (c). Error bars represent median with interquartile range. N.S.: Non-significant.

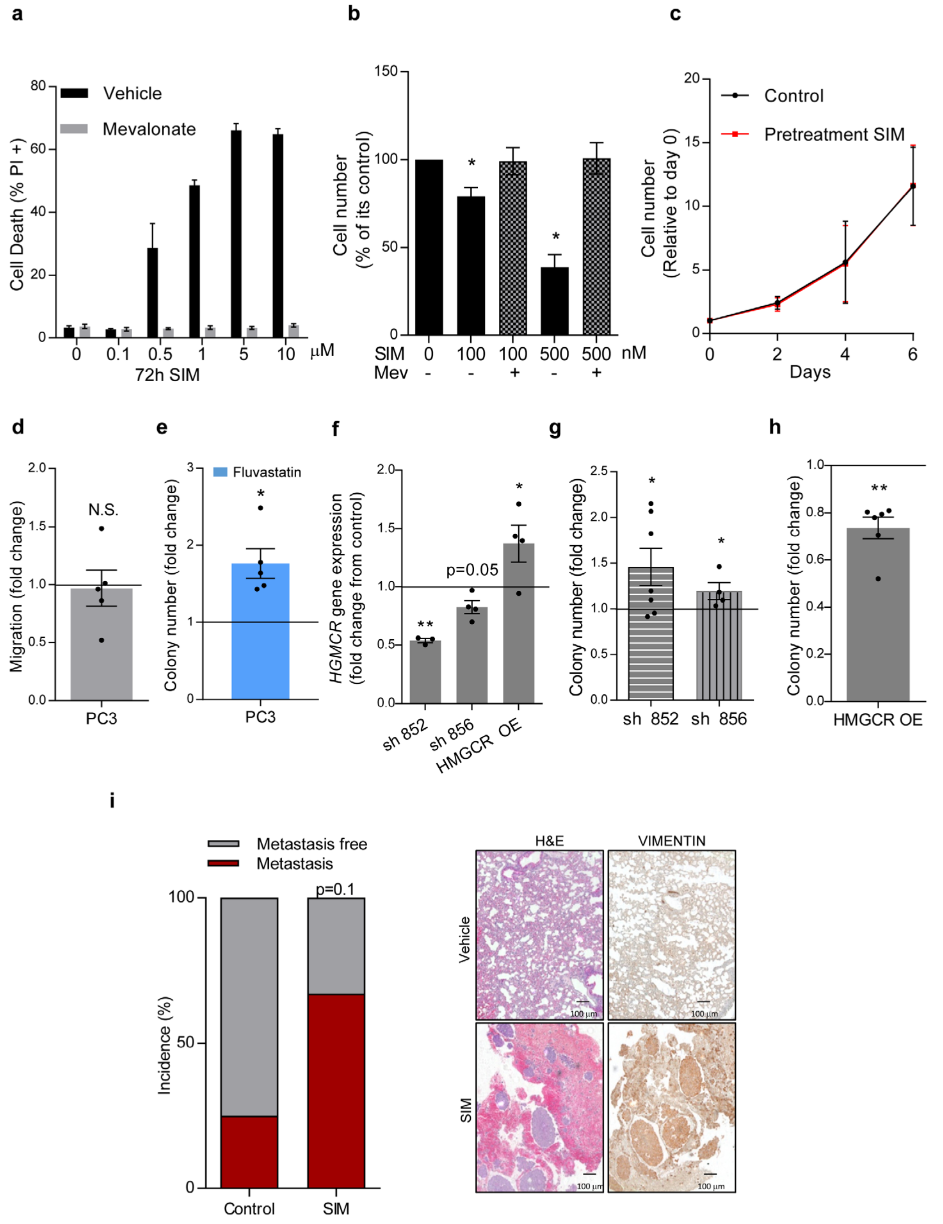

Supplementary Figure 2: (**a**) Propidium iodide (PI) positivity as a readout of cell viability in PC3 cells upon simvastatin (SIM) dose-response treatment (72 hours), in the absence or presence of Mevalonate 500 μM**.** Data is represented as percentage of PI(+) cells (n=3). (**b-c**) Relative cell number quantification upon simvastatin and/or mevalonate administration in PC3 cells (b) or after simvastatin pre-treatment (50 nM) for 7 days (see schematic in Fig. 3b) (c). Data is represented as cell number at day 6 (b) or days 2, 4, and 6 after plating pre-treated cells (c) relative to vehicle (n=3). (**d**) Effect of simvastatin pre-treatment on PC3 cell migration capacity. 50.000 cells were plated in technical triplicates and migration was analyzed after 16h in 0.1% FBS in migration chambers (Corning) with 10% FBS media at the bottom as a chemoattractant (n=5). (**e**) Effect of fluvastatin (n=5) pre-treatment on PC3 cells anchorage-independent growth. (**f**) HMGCR expression by Real time qPCR (using *GAPDH* as housekeeping) in PC3 cells expressing doxycycline-inducible shRNAs (852, n=3, 856, n=4; 100 ng/ml of Doxycycline for 4 days) or ectopic HMGCR (n=4). (**g**) Anchorage independent growth upon HMGCR silencing (sh852 n=7, sh856 n=4). (**h**) Anchorage independent growth of PC3 cells overexpressing HMGCR (n=6). (**i**) Evaluation of metastatic capacity of PC3 cells pre-treated with simvastatin (SIM). $6x10^5$ PC3 cells that had been pre-treated for 7 days with vehicle or SIM were tail vein injected and 10 weeks later mice were sacrificed and checked for metastatic foci in the lung. Left panel shows percentage of mice with metastatic nodules in the lung. Right panel shows histological images of tumor free lung (top) and lung with a metastatic nodule (bottom) stained with H&E (Hematoxylin-eosin) or Vimentin (Control n=8, SIM n=9). Statistical analysis: One sample t test (b, d-h), one sided Fisher's exact test (i). Error bars represent standard error of the mean. N.S: Non-significant. * p<0.05 ** p<0.01.

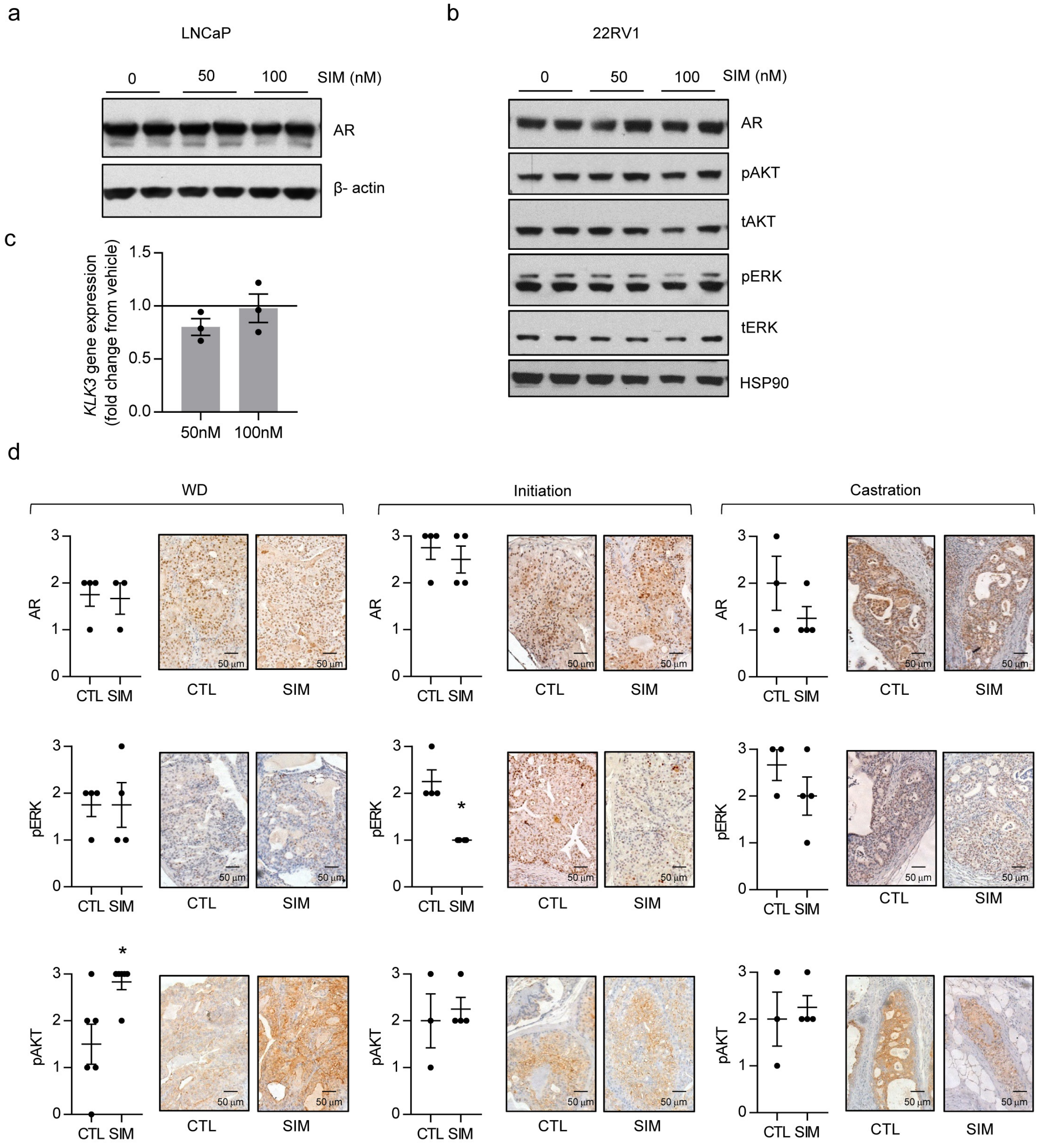

Supplementary Figure 3: (**a, b**) Representative Western blots out of three independent experiments upon treatment with 50 and 100 nM simvastatin (SIM) in LNCaP and 22RV1 cells**.** (**c**) *KLK3* gene expression in LNCaP cells upon 50 and 100 nM simvastatin treatment relative to vehicle. (**d**) Immunohistochemical analysis of AR (androgen receptor), pAKT (serine 473 phosphorylation of AKT) and pERK (tyrosine 202/204 phosphorylation of ERK - extracellular signal regulated kinase). Left, mice fed with western diet (WD) (from Fig 1); middle, *Pten*$^{pc-/-}$ mice at 12weeks of age after 4-weekSIM treatment (from Fig. 2); right, chow or SIM-treated castrated mice (from Supp. Fig. 1). Each panel shows the scoring for each protein expression on the left dot plot, and representative images of the staining from each group of mice fed with simvastatin-loaded (SIM) or chow (CTL) diet. Statistical analysis: One sample t test (c), Mann-Whitney test (d). Error bars represent standard error of the mean. * p<0.05.

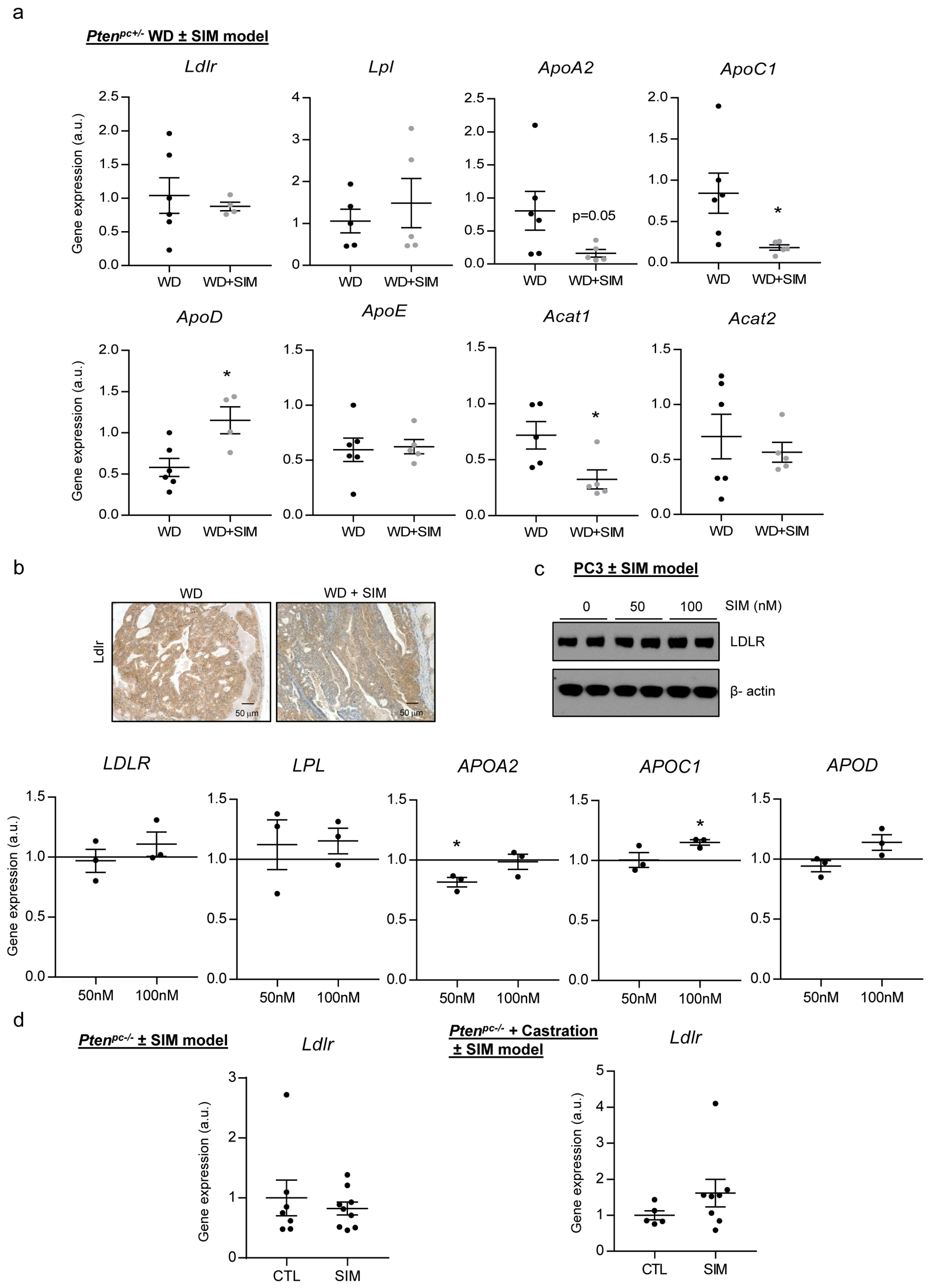

Supplementary Figure 4: (a) Low density lipoprotein receptor (LDLR), lipoprotein lipase (LPL), apolipoproteins (APO) and acetyl CoA cholesterol acyl transferase (ACAT) 1 and 2 gene expression in mice fed with western diet (WD) or WD loaded with simvastatin (WD + SIM) (from Fig 1)**.** (**b**) Representative images of Ldlr immunostaining in mice fed with WD or WD + SIM. (**c**) Representative Western blot (out of three independent experiments) of LDLR expression upon treatment of PC3 cells with 50 and 100 nM simvastatin (SIM) for 72h. (**d**) Expression changes in the indicated genes upon treatment of PC3 cells with 50 and 100 nM simvastatin (SIM) for 72h (n=3). (**e**) *Ldlr* gene expression in prostate-specific *Pten*-deficient (*Pten*$^{pc-/-}$; from Fig. 2) and castrated *Pten*$^{pc-/-}$ mice (from Supplementary Fig. 1) fed with simvastatin-loaded (SIM) diet or chow (CTL). Statistical analysis: Mann-Whitney test (a, e) and One sample T test (d). Error bars represent standard error of the mean. * p<0.05.

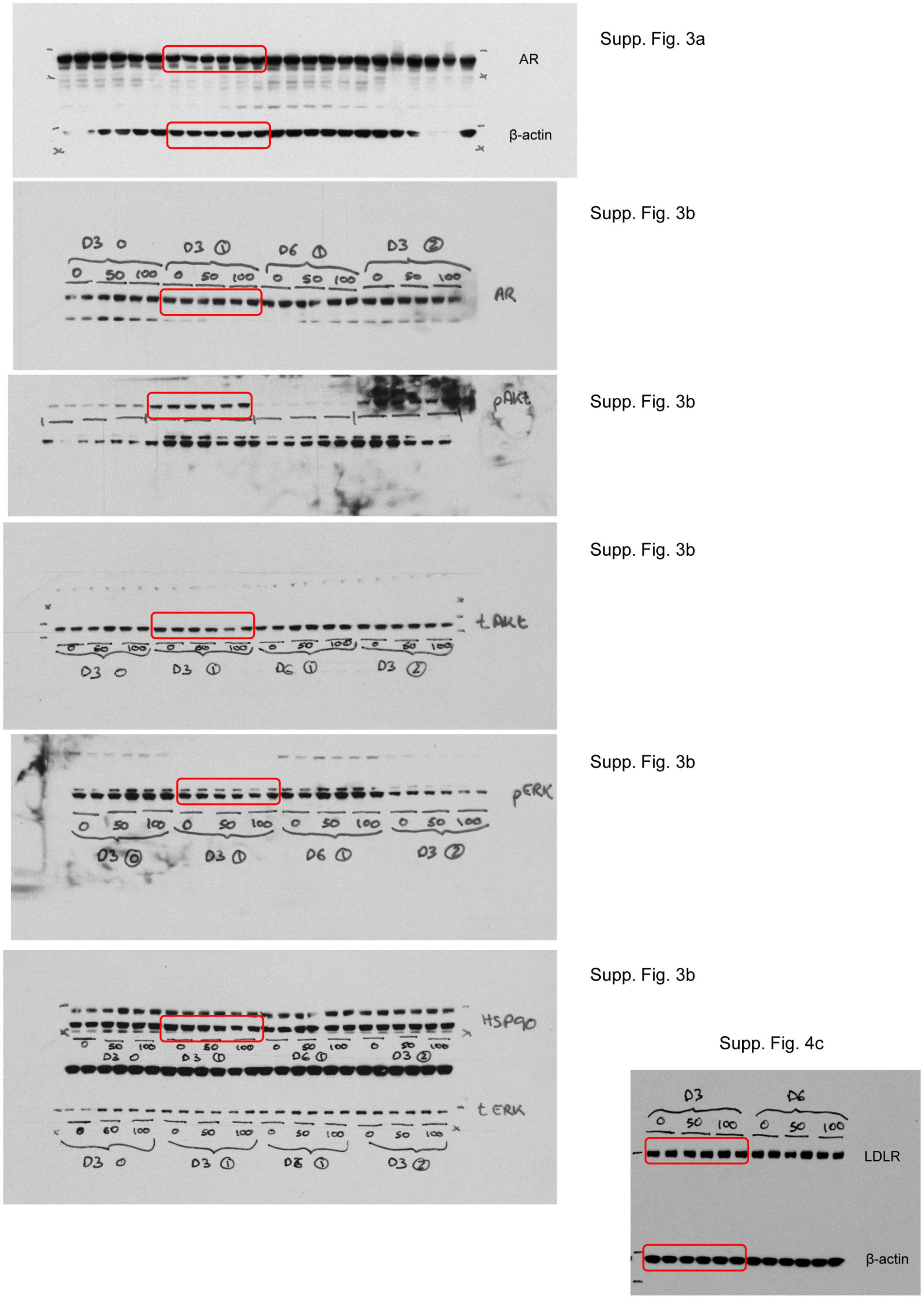

Supplementary Figure 5: Uncropped scans from Supplementary Figures 3 and 4.

For Supplementary Tables 1-5 see in Supplementary Files.

Supplementary Table 1: Summary of reports using statin-treatment *in vivo*.
Supplementary Table 2: Summary of reports using statin-treatment *in vitro*.
Supplementary Table 3: Summary of reports evaluating the association between statin treatment and cancer in human subjects.
Supplementary Table 4: Distribution of low grade (Gleason score ≤ 7) and high grade prostate cancer (Gleason score > 7), according to the treatment with statins.
Supplementary Table 5: Multivariate analysis (Logistic regression analysis) of the association of the indicated chronic treatments with prostate cancer and high grade (Gleason score > 7) prostate cancer.
