# Supplementary material for: Low-dose statin treatment increases prostate cancer aggressiveness
Source: Oncotarget. 2017 Oct 31;9(2):1494–504. doi: 10.18632/oncotarget.22217 (PMC5788577; doi:10.18632/oncotarget.22217)
Supplement: Supplementary file 2 [file oncotarget-09-1494-s002.docx]

**Supplementary Table 1: Summary of reports using statin-treatment *in vivo*.**

| **Author** | **Journal** | **PMID** | **Year** | **Model** | **Statin type** | **Delivery** | **Dose** | **Outcome** |
| --- | --- | --- | --- | --- | --- | --- | --- | --- |
| Newman et al. | JAMA | 8531288 | 1996 | Review rodents | Several | N.A. | Several | Carcinogenesis |
| Minden et al. | Leuk Lymphoma | 11426537 | 2001 | Leukemia trial | Lovastatin | Oral | 80 mg/d | Blast cell growth |
| Masko et al. | Prostate | 27900797 | 2016 | Prostate cancer xenografts | Simvastatin | IP | 11 mg/kg | Tumor growth |
| Schmidmaier et al. | Eur J Haematol. | 17655704 | 2007 | Myeloma trial | Simvastatin | Oral | 80 mg/d | Drug resistance |
| Bjarnadottir et al. | Breast Cancer Res Treat | 23471651 | 2013 | Breast cancer trial | Atorvastatin | Oral | 80 mg/d | Proliferation |
| Inano et al. | Carcinogenesis. | 9328167 | 1997 | Rats radiation carcinogenesis | Simvastatin | Chow | 300 mg/kg | Carcinogenesis |
| Narisawa et al. | Tohoku J Exp Med | 9111762 | 1996 | Colon carcinogenesis | Pravastatin | Water | 1 mg/kg | Carcinogenesis |
| Clutterbuck et al. | Br J Haematol. | 9695968 | 1998 | Leukemia xenografts | Simvastatin | IV | 15 mg/kg | Leukemogenesis |
| Feleszko et al. | Int J Cancer | 10225445 | 1999 | Ras-3T3 xenografts | Lovastatin | IP | 50 mg/kg | Tumor growth |
| Ji et al. | Cancer Prev Res (Phila) | 26908565 | 2016 | Breast cancer trial | Atorvastatin | Oral | 40 mg/d | Tumor growth and molecular markers |
| Stine et al. | Oncotarget | 26503475 | 2015 | Ovarian cancer xenografts | Simvastatin | IP | 3 mg/kg/d | Tumor growth and proliferation |
| Kureishi et al. | Nat Med | 10973320 | 2000 | Angiogenesis | Simvastatin | IP | 0.1 mg/kg/d | Angiogenesis |
| Kochuparambil et al. | J Pharmacol Exp Ther | 21059805 | 2011 | Prostate cancer xenografts | Simvastatin | IP | 4 μg/gbw/d | Growth |
| Babcook et al. | Mol Cancer Ther | 25122066 | 2014 | Prostate cancer xenografts | Simvastatin | Oral | 3.5-7.0 μg/gbw | Tumor growth |
| Chang et al. | Biochim Biophys Acta. | 23583370 | 2013 | Colorectal cancer xenografts | Simvastatin | IP | 20 mg/kg/d | Tumor growth |
