# Supplementary material for: Low-dose statin treatment increases prostate cancer aggressiveness
Source: Oncotarget. 2017 Oct 31;9(2):1494–504. doi: 10.18632/oncotarget.22217 (PMC5788577; doi:10.18632/oncotarget.22217)
Supplement: Supplementary file 3 [file oncotarget-09-1494-s003.docx]

**Supplementary Table 2: Summary of reports using statin-treatment *in vitro*.**

| **Author** | **Journal** | **PMID** | **Year** | **Model** | **Dose** | **Type** | **Outcome** |
| --- | --- | --- | --- | --- | --- | --- | --- |
| Al-Husein et al. | J Cell Physiol. | 23559257 | 2013 | Prostate cancer cell lines | 25 µM | Simvastatin | Adhesion |
| Kureishi et al. | Nat Med | 10973320 | 2000 | Endothelial cells | nM to µM | Simvastatin | Angiogenesis |
| Ingersoll et al. | Cancer Lett | 27687622 | 2016 | Prostate cancer cell lines | 10 µM | Simvastatin | Cell death |
| Stine et al. | Oncotarget | 26503475 | 2015 | Ovarian cell lines | µM | Simvastatin | Cell death |
| Rogers et al. | Anticancer Res | 25862838 | 2015 | Prostate cancer cell lines | ≥ 1 µM | Simvastatin | Cell death |
| Hoque et al. | Cancer Epidemiol Biomarkers Prev | 18199714 | 2008 | Prostate cancer cell lines | µM | Simvastatin - Lovastatin | Cell death |
| Oliveira et al. | J Bioenerg Biomembr | 18679777 | 2008 | Prostate cancer cell lines | ≥ 10 µM | Simvastatin | Cell death |
| Goc et al. | BMC Cancer | 22974127 | 2012 | Prostate cancer cell lines | 25 µM | Simvastatin | Cell death |
| Park et al. | J Urol. | 23085058 | 2012 | Prostate cancer cell lines | ≥ 20 µM | Simvastatin | Cell death |
| Chang et al. | Biochim Biophys Acta. | 23583370 | 2013 | Colrectal cancer cell lines | ≥ 10 µM | Simvastatin | Cell death |
| Newman, A et al. | Leukemia | 7967748 | 1994 | Leukaemic cell lines | 10 µM | Simvastatin | Cell death |
| Menter et al. | PLoS One | 22216116 | 2011 | Various cell lines | 100 nM | Simvastatin -Pravastatin | Cell death |
| Costa et al. | Eur J Pharmacol | 23333250 | 2013 | Prostate cancer cell lines | 60 µM | Simvastatin | Cell death |
| Masko et al. | Prostate | 27900797 | 2016 | Various cell lines | ≥ 100 nM | Simvastatin | Cell death |
| Liang et al. | Sci Rep | 28150753 | 2017 | Breast cancer cell lines | ≥ 4 µM | Simvastatin | DNA replication |
| Brown et al. | Br J Cancer. | 22531631 | 2012 | Prostate cancer cell lines | 1 µM | Several | Invasion |
| Babcook et al. | Mol Cancer Ther | 25122066 | 2014 | Prostate cancer cell lines | µM | Simvastatin | Migration, cell death |
| Aberg et al. | Thromb Haemost. | 18841289 | 2008 | Prostate cancer cell lines | ≥ nM | Simvastatin - Lovastatin | Production of prostasomes |
| Wang et al. | Sci Rep | 27779188 | 2016 | Bladder cancer | µM | Simvastatin | Proliferation |
| Furuya et al. | Prostate Int | 27358845 | 2016 | Prostate cancer cell lines | µM | Simvastatin | Proliferation |
| Olivan et al. | Biomed Res Int. | 25649906 | 2015 | Prostate cancer cell lines | 1 µM | Simvastatin | Proliferation |
| Crosbie et al. | Biomark Res. | 24359683 | 2013 | Leukaemic cell lines | 50 µM | Several | Proliferation |
| Ishikawa et al. | Int J Cancer. | 24346863 | 2014 | Colorectal cancer cell lines | ≥ 5 µM | Simvastatin -Pravastatin | Proliferation |
| Kim et al. | Lipids Health Dis | 24666612 | 2014 | Prostate cancer cell lines | ≥ 50 µM | Simvastatin | Proliferation and cell death |
| Kochuparambil et al. | J Pharmacol Exp Ther | 21059805 | 2011 | Prostate cancer cell lines | 25 µM | Simvastatin | Proliferation and invasion |
