# Supplementary material for: Low-dose statin treatment increases prostate cancer aggressiveness
Source: Oncotarget. 2017 Oct 31;9(2):1494–504. doi: 10.18632/oncotarget.22217 (PMC5788577; doi:10.18632/oncotarget.22217)
Supplement: Supplementary file 4 [file oncotarget-09-1494-s004.docx]

**Supplementary Table 3: Summary of reports evaluating the association between statin treatment and cancer in human subjects.**

| **Author** | **Journal** | **PMID** | **Year** | **Tumor** | **Patient number** | **Study** | **Statin** | **Dose** | **Outcome** |
| --- | --- | --- | --- | --- | --- | --- | --- | --- | --- |
| Sahi et al. | Cancer Epidemiol. | 22683172 | 2012 | Merkel cell carcinoma | 456000 | Prospective | Any | N.A. | Relative risk = 3.16 patients <60 years |
| Matsuzaki et al. | Circulation Journal | 12499611 | 2002 | All | 47294 | Trial 6y | Simvastatin | 5-10 mg | Relative risk (statin treated with low LDL-Cholesterol) = 1.85- 3.16 |
| Chih-Ching et al. | The prostate | 21480313 | 2011 | PrC | 388 | Case-control study | Any | N.A. | Overall Risk = 1.55 |
| Morote et al. | Int J Mol Sci | 25101846 | 2014 | PrC | 2408 | Retrospective | Any | N.A. | Increased risk in High Grade PCa,reduced risk of overall Pca |
| Wettstein et al. | Prostate | 28093792 | 2017 | PrC | 371 | Prospective. Recurrence. | Any | N.A. | No differences with statins. Higher risk low LDL |
| Pottegård et al. | Eur Urol | 26603781 | 2016 | Renal c c | 4606 | Case-control | Any | N.A. | Overall Risk = 1.25 in women |
| Tan et al. | Asian J Androl | 27924788 | 2016 | PrC | 104707 | Meta-analysis | Any | N.A. | No differences |
| Friedman et al. | Pharmacoepidemiol Drug Saf | 17944002 | 2008 | All | 361859 | Prospective | Lovastatin and simvastatin | N.A. | No differences |
| Mamtani et al. | PLoS Med | 27116322 | 2016 | Colon | 22163 | Meta-analysis | Any | N.A. | No differences |
| Sun et al. | Lipids Health Dis | 26526340 | 2015 | All | 5379 | Meta-analysis | low LDL + Any statin | N.A. | No differences |
| Lv | Pharmacol Res | 24602799 | 2014 | All | 47296 | Meta-analysis ≥ 6y | Any | N.A. | No differences |
| Bonovas et al. | Int J Cancer | 18491405 | 2008 | PrC | 880000 | Meta-analysis | Any | N.A. | No differences in total. Protection in advanced Pca |
| Bonovas | Drugs | 25288321 | 2014 | All | N.A. | Review of meta- analysis | Any | N.A. | No differences except with pravastatin in elderly patients. |
| Olivan et al. | Biomed Res Int | 25649906 | 2015 | PrC | 2408 | Epidemiology | Any | N.A. | Protective in total PCa statins + aspirin, neutral only statins, increase HGPC statins + aspirin |
| Chen et al. | Biomed Res Int. | 27975064 | 2016 | Ovarian | 60 | Retrospective | Any | N.A. | No differences |
| Ford et al. | Circulation | 26864092 | 2016 | All | 6595 | 20 ys follow up | Pravastatin | 40 mg | No differences |
| Jespersen et al. | Cancer Epidemiol | 24275259 | 2014 | PrC | 42480 | Case-control | Any | N.A. | No differences |
| VVAA | Lancet | 7968073 | 1994 | All | 4444 | Random Trial | Simvastatin | N.A. | No differences in malignancies |
| Danzig et al. | Prostate Cancer Prostatic Dis. | 25403419 | 2015 | PrC | 767 | Retrospective | Any | N.A. | No differences |
| Allott et al. | Cancer Epidemiol Biomarkers Prev | 26819265 | 2016 | PrC | 1930 | Retrospective | Any | N.A. | Protective |
| Bansal et al. | PLoS One | 23049713 | 2012 | PrC | 56847 | Meta-analysis | Any | N.A. | Protective |
| Huang et al. | J Natl Cancer Inst | 28040693 | 2016 | Pancreas | 2142 | Retrospective | Any | N.A. | Protective |
| McGlynn et al. | Cancer Epidemiol | 25113938 | 2014 | HCC | 94 | Case-control | Any | N.A. | Protective, OR= 0.32 |
| Allott et al. | BJU Int | 24588774 | 2014 | PrC | 1146 | RP patients postoperative | Any | N.A. | Reduced risk biochemical recurrence |
| Krane et al. | J Urol | 19913252 | 2010 | PrC | 3828 | Retrospective review | Any | N.A. | Protective |
| Platz et al. | J Natl Cancer Inst | 17179483 | 2006 | PrC | 34989 | Prospective | Any | N.A. | Protective on advanced and metastatic PCa. No differences in overall population. |
