# Supplementary material for: Low-dose statin treatment increases prostate cancer aggressiveness
Source: Oncotarget. 2017 Oct 31;9(2):1494–504. doi: 10.18632/oncotarget.22217 (PMC5788577; doi:10.18632/oncotarget.22217)
Supplement: Supplementary file 5 [file oncotarget-09-1494-s005.docx]

**Supplementary Table 4: Distribution of prostate cancer patients according to the tumor grade and statins treatment**.

| Prostate cancer grade^1^ | No statins | Statins | Total |
| --- | --- | --- | --- |
| Low grade (%) | 416 (67.5) | 135 (58.2) | 551 (65.0%) |
| High grade (%) | 200 (32.5) | 97 (41.8) | 297 (35.0%) |
| Total | 616 (72.6%) | 232 (37.4%) | 848 (100%) |

^1^ Low grade (Gleason score <7), High grade (Gleason score 8-10)
