# Supplementary material for: Low-dose statin treatment increases prostate cancer aggressiveness
Source: Oncotarget. 2017 Oct 31;9(2):1494–504. doi: 10.18632/oncotarget.22217 (PMC5788577; doi:10.18632/oncotarget.22217)
Supplement: Supplementary file 6 [file oncotarget-09-1494-s006.docx]

**Supplementary Table 5: Multivariate analysis (Logistic regression analysis) of the association of the indicated chronic treatments with prostate cancer and high grade (Gleason score > 7) prostate cancer**.

| Predictor | Prostate cancer | | High grade prostate cancer | |
| --- | --- | --- | --- | --- |
|  | OR (95%CI) | p Value | OR (95% CI) | p Value |
| Age, years | 1.046 (1.033-1.059) | 0.001 | 1.112 (1.082-1.143) | 0.001 |
| Serum PSA, ng/dL | 1.042 (1.030-1.053) | 0.001 | 1.042 (1.030-1.053) | 0.001 |
| DRE1, abnormal vs normal | 1.724 (1.238-2.130) | 0.001 | 1.629 (1.215-1.936) | 0.001 |
| Statins, yes vs no | 0.692 (0.563-0.852) | 0.001 | 1.502 (1.010-2.234) | 0.044 |
| IECAs, yes vs no | 1.011 (0.824-1.239) | 0.920 | 1.174 (0.796-1.732) | 0.418 |
| Metformin, yes vs no | 0.775 (0.577-1-041) | 0.091 | 0.393 (0.203-0.761) | 0.007 |

1 digital rectal examination.
